# Antimicrobial and immunomodulatory efficacy of extracellularly synthesized silver and gold nanoparticles by a novel phosphate solubilizing fungus *Bipolaris tetramera*

**DOI:** 10.1186/s12866-015-0391-y

**Published:** 2015-02-27

**Authors:** Faria Fatima, Preeti Bajpai, Neelam Pathak, Sarika Singh, Shivam Priya, Smita Rastogi Verma

**Affiliations:** Department of Biosciences, Integral University, Lucknow, 226026 India; Division of Toxicology, Central Drug Research Institute, Lucknow, 226031 India; Division of Environmental Carcinogenesis, Indian Institute of Toxicology Research, Lucknow, 226026 India; Department of Biotechnology, Delhi Technological University, Delhi, 110042 India

**Keywords:** *Bipolaris tetramera*, Cytotoxicity, Gold nanoparticle, Immunomodulatory, Silver nanoparticle

## Abstract

**Background:**

Particulates of nanometers size have occupied a significant area in the field of medicinal and agricultural purposes due to their large surface-to-volume ratio and exceptional physicochemical, electronic and mechanical properties*.* Myconanotechnology, an interface between mycology and nanotechnology is budding nowadays for nanoparticle-fabrication using fungus or its metabolites. In the present study, we have isolated and characterized a novel phosphate solubilizing fungus *B. tetramera* KF934408 from rhizospheric soil. This phosphatase releasing fungus was subjected to extracellular synthesis of metal nanoparticles by redox reaction.

**Results:**

Silver (AgNPs) and gold nanoparticles (AuNPs) were characterized by dynamic light scattering and transmission electron microscopic analysis. The formulated AgNPs were irregular shaped with a size ranging between 54.78 nm to 73.49 nm whereas AuNPs were spherical or hexagonal, with a size of 58.4 and 261.73 nm, respectively. The nanoparticles were assessed for their antibacterial and antifungal efficacy. The results showed effective antimicrobial activity of AgNPs against *Bacillus cereus, Staphylococcus aureus, Enterobacter aeroginosa* and *Trichoderma sp*. at higher concentrations, however, AuNPs possessed only moderate antibacterial efficacy while they found no antifungal activity. Cytotoxicity analysis of nanoparticles on *J774* and *THP1 α* cell lines revealed the dose dependence in case of AgNPs, while AuNPs were non-toxic at both low and high doses. Furthermore, significant elevation of intracellular ROS was observed after 4 h of incubation with both the nanoparticles. The capping of fungal proteins on the particulates might be involved in the activities demonstrated by these inert metal nanoparticles.

**Conclusion:**

In conclusion, the findings showed that the metal nanoparticles synthesized by fungus *B. tetramera* could be used as an antimicrobial agents as well as cost effective and nontoxic immunomodulatory delivery vehicle.

**Electronic supplementary material:**

The online version of this article (doi:10.1186/s12866-015-0391-y) contains supplementary material, which is available to authorized users.

## Background

Myconanotechnology is an emerging field, where fungi can be harnessed for the synthesis of nanomaterials or nanostructures with desirable shape and size. Potential applications of myconanotechnology have fascinated microbiologists and other researchers to contribute in providing incremental solutions through green chemistry approaches for targeted drug delivery. Nanosized particles have attracted worldwide attention due to their specific properties and applications in areas such as biomedical sciences [1].

In order to meet the wide scope of nanomaterials, an overwhelming number of protocols have been exploited for their synthesis, but unfortunately, most of them are capital-intensive, inefficient in material and energy use, and often pose health hazards because of usage of toxic chemicals [2-4]. Often chemical synthesis leads to the adsorption of toxic chemical species on the surface resulting in undesirable impact on human health [5]. Therefore, there is a persistent need to develop ecofriendly methods for the preparation of nanoparticles with better applicability and limited toxicity. On the other hand, synthesis using bio-organisms (bacteria, fungi, and plant extracts) is compatible with the green chemistry principles [6]. The biosynthesized nanoparticles, are eco-friendly and biocompatible for pharmaceutical applications. Biosynthesis of nanoparticles is a kind of bottom up approach where the main reaction is the reduction/oxidation reaction. The microbial enzymes possessing high redox potential are usually responsible for the reduction of metal compounds into their respective nanoparticles. As a result, nanomaterial synthesis mediated by biological systems has attracted scientific interest across the globe.

Fungi are preferred microbes for nanostructure formulation due to their ability to produce high amount of secretory proteins [7]. Numbers of reports have showed the biological synthesis of metal nanoparticles but the potential of a phosphate solubilizing fungi *B. tetramera* has not yet been demonstrated. The present study was performed to synthesize the silver and gold nanoparticles from *B. tetramera* (GenBank: KF934408)*,* a novel phosphate solubilizing fungus*,* isolated from rhizospheric region. The potential antimicrobial, cytotoxic and immunomodulatory activity of these myconanoparticles synthesized has also been evaluated.

## Methods

### Isolation, screening and characterization of *B. tetramera* (KF934408)

The fungus was isolated from rhizospheric soil of Kukrail forest, Lucknow, India and screened by its phosphate solubilizing ability on Pikovskaya’s medium by plate assay [8]. The fungal colonies were subculture on fresh petriplates containing media and the plates were incubated in inverted position for 72 h at 28 ± 2°C and further screened by observing halo zones around the colony.

Biochemical characteristics of the fungus were tested on the basis of lacto-colony morphology, microscopic analysis of conidia, phosphate solubilization index, starch hydrolysis test and cellulose hydrolysis test according to standard protocols [9].

The fungus was further characterized at molecular level by 18S rRNA sequencing. Genomic DNA was isolated from pure culture of the isolated fungus and subjected to high–fidelity PCR using universal primers *i.e.* forward primer (5’-GGAAGTAAAAGTCGTAACAAGG-3’) and reverse primer (5’-TCCTCCGCTTATTGATATGC-3’) and analyzed on 1% agarose gel. The PCR product was sequenced bi-directionally using the forward and reverse primers. Homology between this 18S rRNA sequence and the strains available at the public databases (Genbank, EMBL and DDBJ) was determined using BLASTN sequence match routines. The UPGMA (Unweighted Pair Group Mathematical Average) algorithm was used to perform hierarchical cluster analysis [10]. The sequences were aligned using CLUSTALW2 program and its phylogenetic and molecular evolutionary analysis were conducted. Sequences analysis was performed by alignment of the partial 18S rRNA gene sequences to those obtained from the GenBank database, using the program BLAST (NCBI BLAST® homepage). The nucleotide sequences of 18S rRNA gene segments, determined in this study have been deposited in GenBank database under accession number KF934408.

### Myco-synthesis of metal nanoparticles

The isolated fungus was grown aerobically in MGYP (Maltose glucose yeast peptone) broth comprising of malt extract (0.5%), glucose (1%), yeast extract (0.3%) and peptone (0.5%). The culture was incubated at 27°C and harvested after 120 h of growth by sieving through a plastic sieve followed by extensive washing with sterile double-distilled water. Initially, 15 g of biomass (wet weight) was transferred to 100 ml deionized water for 48 h at 27°C in an Erlenmeyer flask and agitated at 150 × rpm for release of secretory proteins. 1 mM of silver nitrate and gold tetra hydrate were added to the Erlenmeyer flasks and the reaction was allowed to proceed in dark for synthesis of silver nanoparticles (AgNPs) and gold nanoparticles (AuNPs), respectively. Time-dependent formation of silver and gold nanoparticles was observed by using ultraviolet-visible spectrophotometer (Beckman DU-20 spectrophotometer). The scanning range was 350-650 nm for AgNPs and 400-800 nm for AuNPs at a scan speed of 420 nm/min. The data was recorded and analyzed using “UVWinlab” software.

### Differential light scattering (DLS)

The suspensions of AgNPs and AuNPs were prepared in distilled water (dH_2_O) by using a bath-sonicator (ULTRAsonik 57 X, 50/60 Hz, California, USA) prior to size measurements. Viscosity measurements were performed on dH_2_O with the aid of a Viscometer SV-10 (A&D Instruments Ltd., UK) at 25°C and the recorded values were used in all DLS size estimations. The viscosity of dH_2_O at 25°C was 0.887 centipoise. DLS size measurements were performed with the aid of a Malvern Zeta Sizer Nano ZS (Malvern Instruments, Worcestershire, UK) operating with version 5.03 of the systems Dispersion Technology Software (DTS Nano). The samples for DLS were equilibrated at 25°C for 3 min before each measurement. The refractive index (RI) of AgNP.dH_2_O was 1.330 and that for AuNP.dH_2_O was 1.430.

### Transmission electron microscopy (TEM)

The synthesized AuNPs and AuNPs were characterized by TEM studies. The samples for TEM analysis were prepared by placing a drop of synthesized nanoparticles over gold coated negative grid followed by evaporation of the solvent [11]. TEM analysis was performed on Perkin-Elmer model, which was operated at an accelerating voltage of 1000 kV.

### Antibacterial efficacy

Pure cultures of five pathogenic bacteria, namely, *Bacillus subtilis, Bacillus cereus, Staphylococcus aureus, Escherichia coli* and *Enterobacter aeroginosa* were procured from National Chemical Laboratory, Pune. Bacterial stock cultures were maintained at 4°C on nutrient agar media slants and subcultured on nutrient broth media for antibacterial analysis of NPs. The turbidity (OD_600_) of inocula was maintained at 0.8 corresponding to ~1 × 10^8^ CFU/ml. The antibacterial potential of both the nanoparticles was analyzed using agar well diffusion method [12]. 5 mm wells of Muller Hinton agar in diameter were prepared and filled with a range of concentrations (10 μM, 50 μM, 100 μM, 150 μM, Ab, Ab + (Ag/Au)NP ). Each experiment was performed in triplicate and the average zone of inhibition, excluding well, for each case was recorded. 1 mM AgNO_3_ and HAuCl_4_ were used as negative controls for analyses of AgNPs and AuNPs, respectively.

### Antifungal efficacy

The antifungal activity of AgNPs and AuNPs was tested against *Aspergillus niger* and *Trichoderma* sp. by agar well diffusion method [13]. Aliquot of 50 μl spores suspension (1 x 10^8^ spores/ml) of each isolate was streaked in radial patterns on the surface of media plates. 5 mm wells in diameter were prepared and filled with a range of concentrations*,* (150 μM, 200 μM and 250 μM) of both the nanoparticles prepared. The cultured plates were incubated at 28 ± 4°C for 7 days. The average inhibition zone, excluding well, for each case was measured.

### Maintenance of cell lines

Mouse macrophage cell line *J774* and human macrophage cell line *THP1 α* was procured from the National Center of Cell Sciences, Pune, India and maintained at Animal Tissue Culture facility of Central Drug Research Institute (CDRI). Cells were maintained in Dulbecco's modified Eagle's medium (DMEM) supplemented with 10% Foetal calf serum (FCS) and 1% antibiotic-antimycotic solution, at 37°C and 5% CO_2_ using standard cell culture methods.

### Cytotoxicity assay

Cell viability was determined by MTT [3-(4, 5-dimethylthiazol-2-yl)-2, 5- diphenyltetrazolium bromide] conversion assay [14]. The 1 × 10^6^ cells/ml were plated in 96-well culture plates and incubated with increasing concentrations of nanoparticles (10 μM, 25 μM, 50 μM, 100 μM, 150 μM and 200 μM) for 24 h at 37°C in CO_2_ incubator. The MTT dye was added to each well and plate was incubated at 37°C for 4 h. The absorbance of insoluble formazan salts was assessed at 550 nm using Powerwave XS “BIOTEK, USA” spectrophotometer [15]. Data produced were used to plot a dose-reaction curve and the concentration of these metal nanoparticles required to kill 50% of cell population (IC_50_) was determined.

$$ \mathrm{Cell}\ \mathrm{viability}\ \left(\%\right)\kern0.5em  = \frac{\mathrm{Mean}\ \mathrm{O}\mathrm{D}\ }{\mathrm{Control}\ \mathrm{O}\mathrm{D}} \times 100 $$

### Intracellular reactive oxygen species estimation

Intracellular oxidative stress was estimated by using fluorescent dye 2’, 7’-dichlorofluorescin di-acetate (DCFH-DA), a well accepted fluorescent marker for study of intracellular hydroperoxides [16]. The experiment was performed according to the protocol described by Goswami et al. [17] with slight modifications. Primarily, healthy confluent cells were harvested and seeded (1000 cells/well) into black bottomed 96 well plates (Nunc, Denmark) and allowed to adhere for a period of 24 h prior to exposure. For ROS quantification, both mouse and human macrophage cells were plated and distributed in triplicates. A working stock of 20 μM DCFH-DA in phosphate buffered saline (PBS) was prepared and all test concentrations, unexposed negative controls and positive controls were prepared and exposed to the cells in this working stock. The negative control consisted of the 20 μM DCFH-DA solution in PBS, whereas the positive control consisted of 1 μM hydrogen peroxide (H_2_O_2_) in 20 μM DCFH-DA/PBS solution. The test concentrations consisted of a continuous range of AgNPs and AuNPs. Lipopolysaccharide (1 μg/ml) was used as a mitogen for stimulation of macrophages as well as comparison of phagocytic activity of stimulated and non-stimulated macrophages. The test concentration for AgNPs/AuNPs was 10 μg/ml and the incubation period ranged from 2 h to 6 h. The rate of intracellular oxidative stress was monitored by measuring their fluorescence intensity *via* fluorometer “BIOTEK-FLX800 USA” emission at 520 nm (by 485 nm excitation).

### Statistical analysis

All the experiments were conducted in triplicates and results were expressed as mean ± SD. One-way analysis of variance (ANOVA) with a Dunnett’s test was performed for the multiple comparisons for normally distributed samples with homogenous variance. Statistically significant differences were set at p < 0.05.

## Results

### Screening and fungal characterization

**Figure 1:**
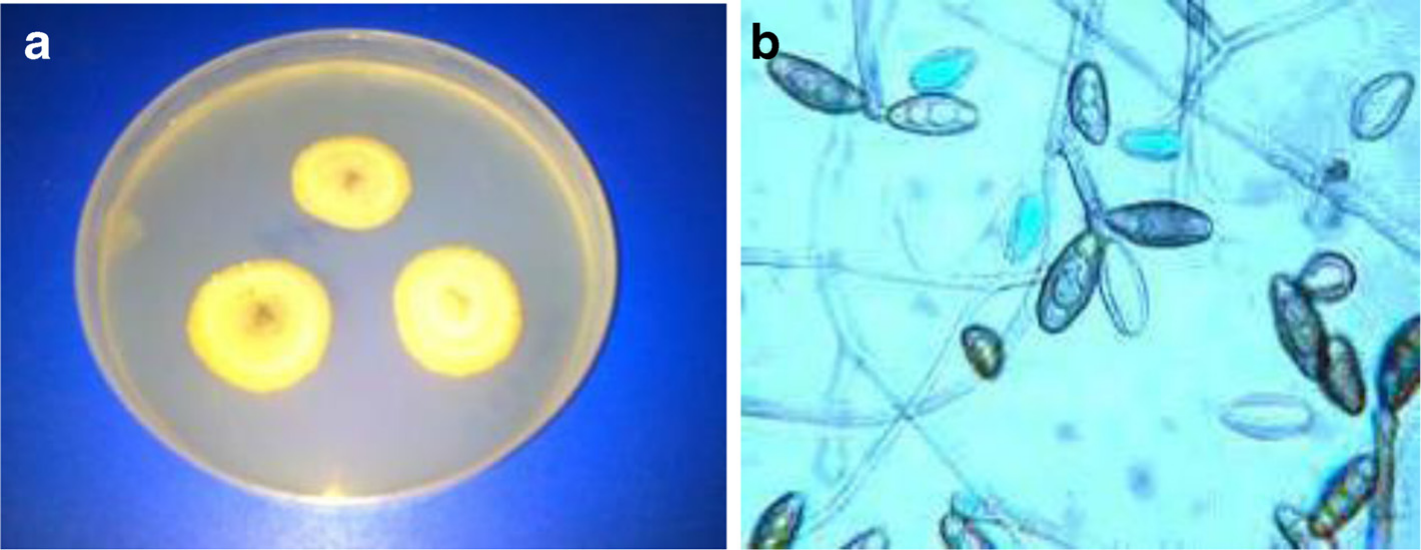
**Morphological features of** ***Bipolaris tetramera*** **(a) Sabouraud agar plate demonstrating colonies of** ***Bipolaris tetramera*** **(b) Microscopic analysis of** ***Bipolaris tetramera*** **after lactophenol staining.**

After successive serial dilutions of rhizospheric soil, all the fungi were checked on the Pikovskaya’s media for their phosphate solubilization efficiency. On Pikovskaya’s medium, the transparency of the media is a primary indicator of phosphate solubilization to be visualized as halo formation in plate assay. Based on plate assay thirty-two fungal were isolated from soil samples. Among these isolated 32 fungal, 20 fungal showed the potent phosphate solubilizing activity. Among all 20, the most promising fungus isolate was FUK 29. This fungus was characterized morphologically, biochemically and at molecular level. The solubilization index of selected fungus was recorded as 3.8 cm. Further, colony morphology was analyzed on the Sabouraud agar medium (Figure 1a) and conidial pattern was analyzed microscopically after lactophenol staining (Figure 1b). Moreover, this fungus gave a negative test for starch hydrolysis while a positive test for cellulose degrading ability.

For further characterization, the genomic DNA was amplified using 18S rRNA specific primers (ITS 4 and ITS 5). An amplicon of 520 bp was observed on 1% agarose gel. The DNA sequencing of the amplified product and its BLAST analysis confirmed the fungus as *B. tetramera* (KF934408). The phylogenetic analysis (Additional file 1) revealed the fungus to belong to Pleosporacae family and the genus was *Bipolaris* (Details appended in Additional file 1).

### Synthesis and size estimation of silver and gold nanoparticles

Two types of nanoparticles were synthesized from *B. tetramera.* The fungal filtrate was used as reducing and stabilizing agent for 1 mM of silver nitrate and gold tetrahydrate salts. The appearance of a brownish color in solution gave the clear indication of the formation of AgNPs in the reaction mixture while production of AuNPs was confirmed by the color change from yellow solution to pinkish violet. The color change is due to the surface plasmon resonance exhibited by the metal nanoparticles. 1 mM AgNO_3_/HAuCl_4_ that act as control when subjected to similar conditions did not demonstrate any color change.

### Ultraviolet-visible spectrophotometric analysis

Ultraviolet-visible spectrophotometer showed no evidence of absorption in the range of 400-800 nm for the fungal extract while the fungal extract exposed to AgNO_3_ and HAuCl_4_ showed a distinct absorption at around 350 nm and 650 nm, with a peak at 380 nm for AgNPs (Additional file 1) and 570 nm for AuNPs (Additional file 1), respectively.

### Dynamic light analysis (DLS)

DLS spectra showed an intensity of 109.4 nm for AgNPs (Additional file 1) and 73.82 nm for AuNPs (Additional file 1). The size variation is due to oxidation of metal salts into their respective nanoparticles in the presence of enzymes. This technique enables the particle size determination by measuring the random changes in the intensity of light scattered from a suspension or solution.

### Transmission electron microscopic (TEM) analyses

**Figure 2:**
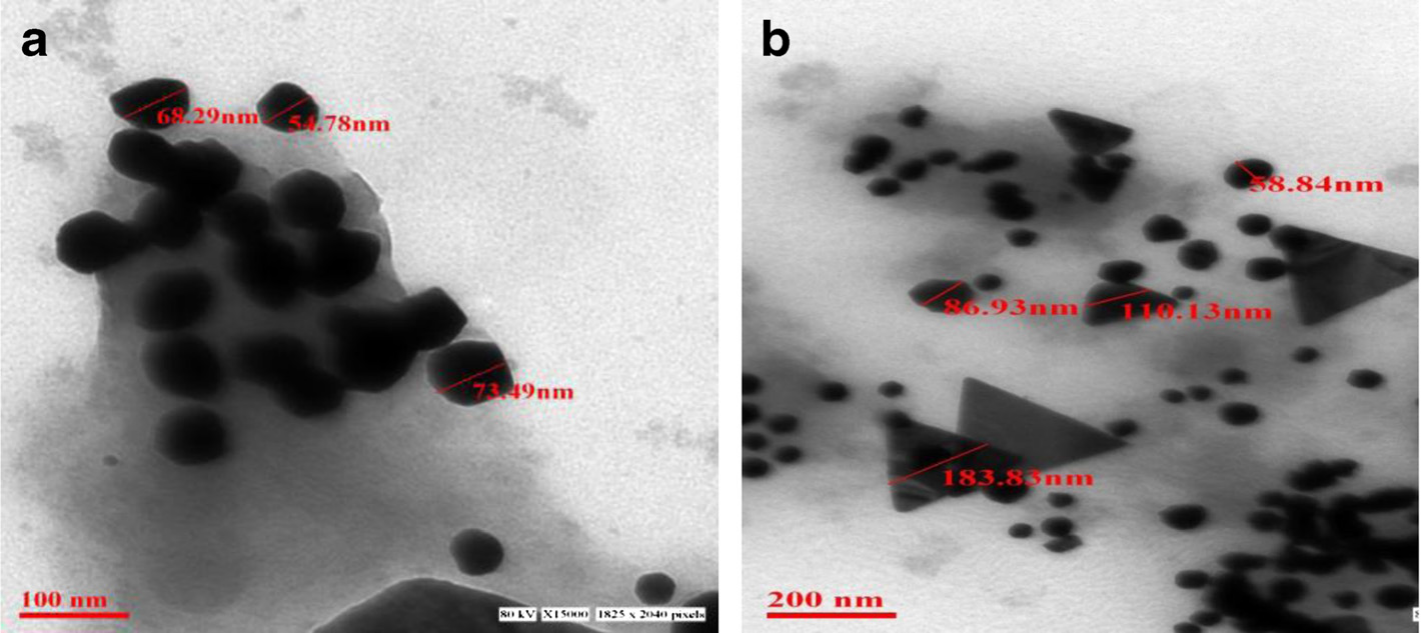
**Transmission electron microscopic analysis of nanoparticles. (a)** AgNPs; **(b)** AuNPs.

TEM micrographs of AgNPs depicted spherical nanoparticles while AuNPs showed three different forms *viz.,* spherical, triangular and hexagonal. This was further confirmed by the representative images recorded from the drop-coated film of the silver/gold nanoparticles uniformly dispersed on grid. The size of the silver AgNPs ranged between 54.78 nm to 73.49 nm (Figure 2a) whereas that of AuNPs ranged from 58.4 nm (spherical), 110.13 nm (triangular) and 261.73 nm (hexagonal), respectively (Figure 2b).

### Antibacterial activity of AgNPs and AuNPs

**Figure 3:**
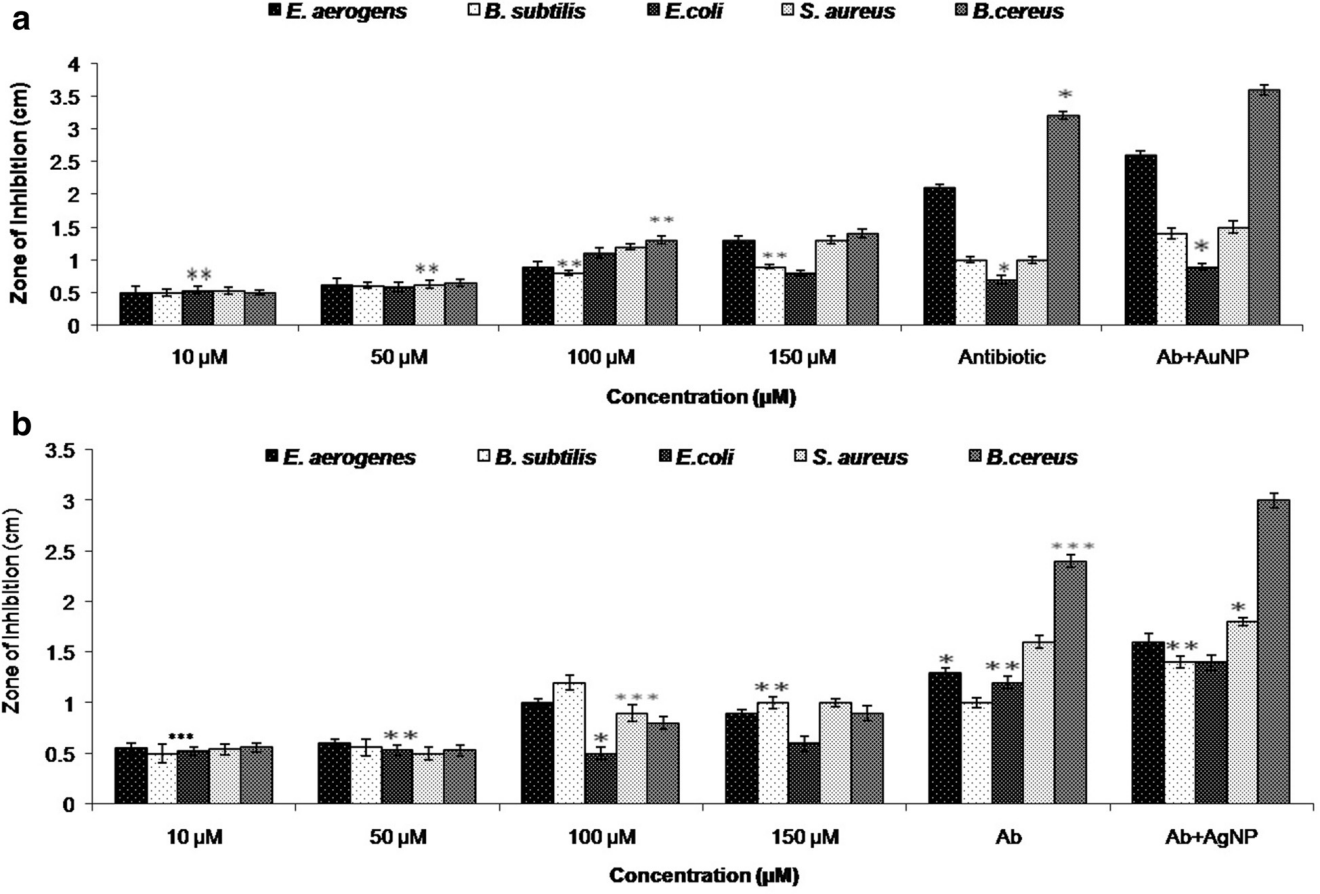
**Comparative analysis of antibacterial activity of nanoparticles (a) AgNP; (b) AuNP for different pathogenic bacteria.** Results are presented in relative units compared with controls (Ab). Data represents the mean ± standard deviation of three individual experiments p < 0.05.

The bactericidal activity of both the nanoparticles were studied using the pathogenic strains of bacteria, namely *B. subtilis, B. cereus, S. aureus, E. coli* and *E. aerogenes* using agar well diffusion method. After the incubation time, zone of inhibition (clear zones) were observed against all the test organisms with both AgNPs and AuNPs. The results recorded in centimetres for AgNPs and AuNPs are shown in Figure 3a and b, respectively.

The comparative histogram demonstrated that the best antibacterial efficacy of AgNP was against *B. cereus* followed by *S. aureus.* Marked increase in antibacterial activity was demonstrated with increasing concentration of AgNPs (Figure 3a). In addition, the efficacy of AgNP was found to be enhanced in combination with the antibiotic tetracycline rather than alone. Similar results were observed with the AuNPs (Figure 3b).

### Antifungal activity of AgNPs and AuNPs

**Figure 4:**
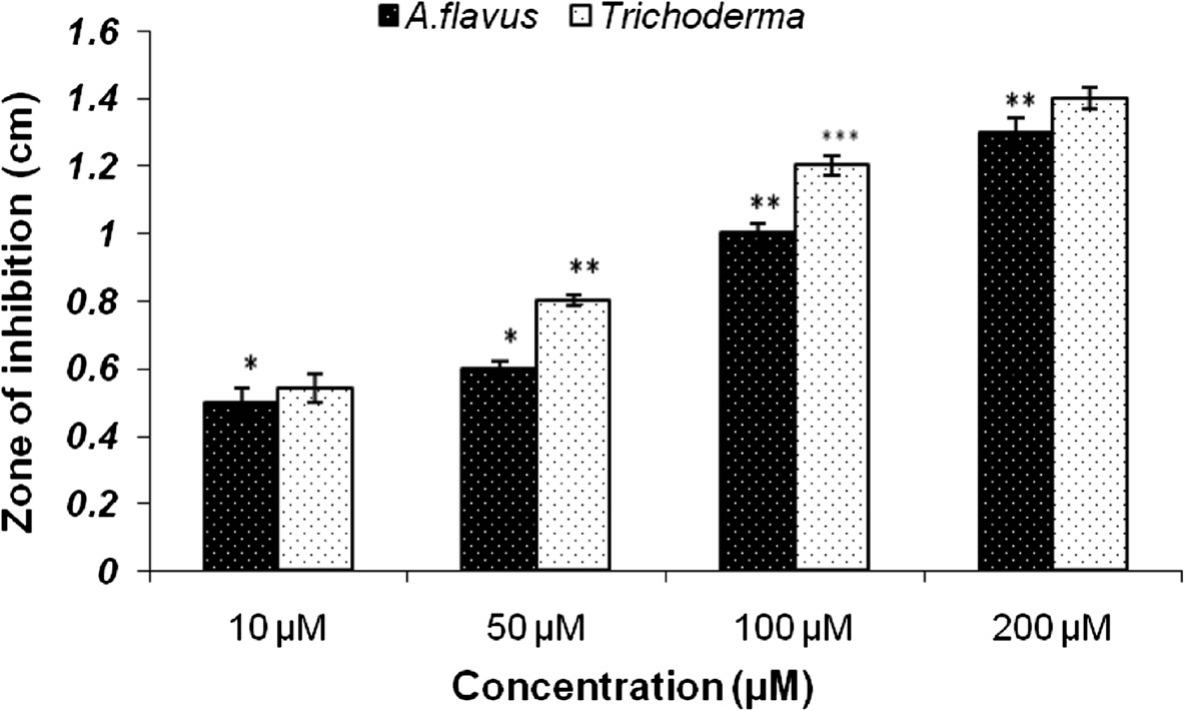
**Histogram showing antifungal activity of AgNP.** Results are presented in relative units compared with controls. Data represent the mean ± standard deviation of the mean of three individual experiments. p < 0.05.

The colloidal AgNPs inhibited the growth of the fungus (*Aspergillus niger* and *Trichoderma)* which was seeded in the Muller Hinton agar plate and formed a zone of inhibition around the central cavity. The zone of inhibition with diameter of 1 cm was recorded in case of *Aspergillus niger* and 1.2 cm in *Trichoderma* (Figure 4) where as no zones of inhibition were found in case of AuNPs, thereby indicating that AuNPs did not have any antifungal activity (data not shown).

### *In vitro* cytotoxicity assay

**Figure 5:**
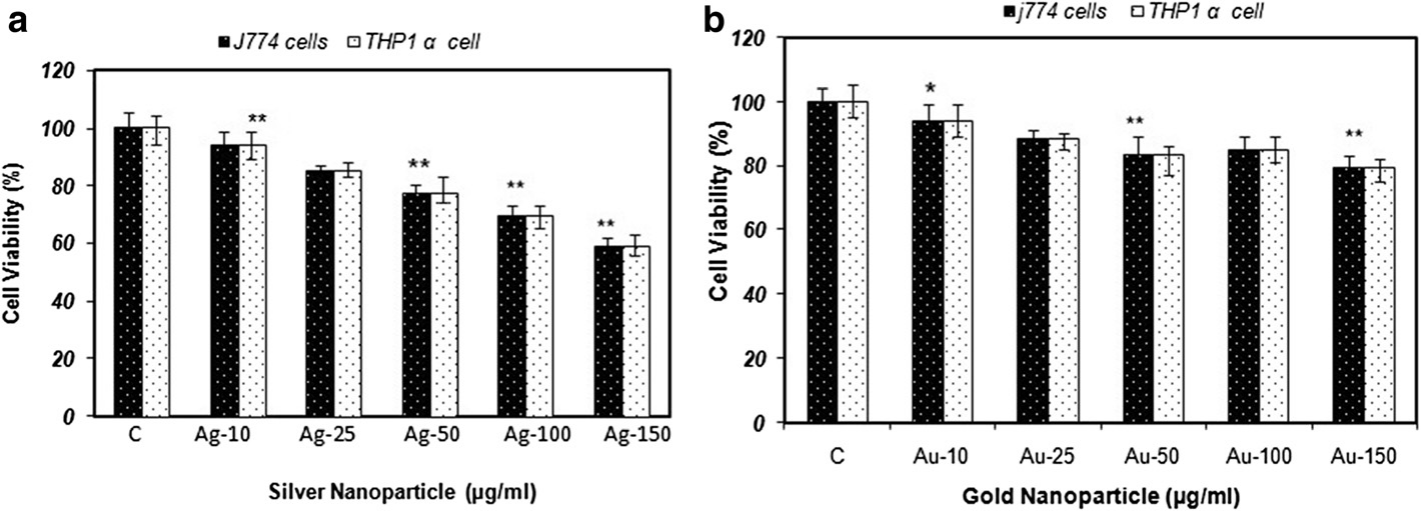
**Dose-dependent effect of nanoparticles (a) AgNPs; (b) AuNPs over cell viability using MTT assay on** ***J774*** **and** ***THP1 α*** **cells.** Results are presented in relative units compared with controls. Data represent the mean ± standard deviation of the mean of three individual experiments. p < 0.05.

To determine if nanoparticles are appropriate biomaterials that produce no toxic effects, cell viability and cytotoxicity assays were performed. The results indicated significant cytotoxicity effects of nanoparticles in a dose dependent manner. These nanoparticles depicted dose-dependent cytotoxicity to *J774* and *THP1 α* cell lines. At low doses (10 μg) no cytotoxic effects were observed whereas at high doses of 100-150 μg mild cytotoxicity effects were observed which might be due to over-accumulation of metal nanoparticles inside the cell (Figure 5a and b).

### ROS level

**Figure 6:**
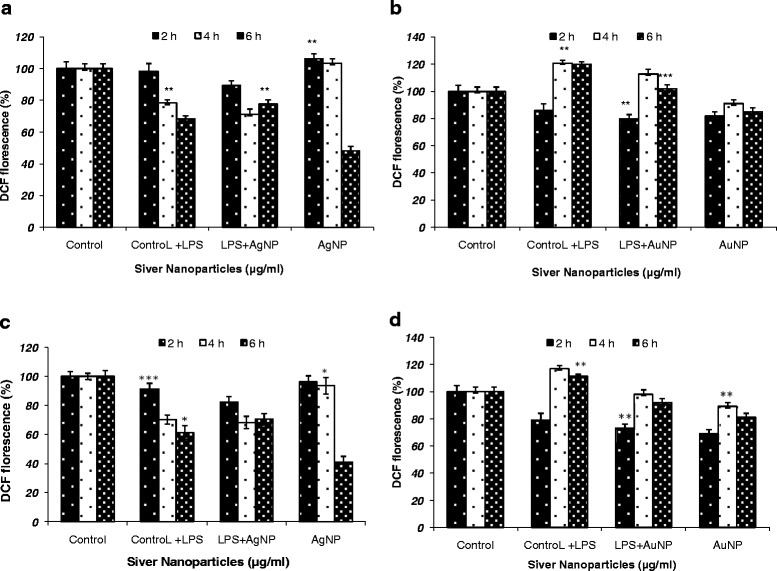
**ROS estimation in** ***J774*** **cell lines (a and b) and** ***THP1 α*** **cell lines (c and d) after incubation with nanoparticles (1 & 3 AgNPs; (2 & 4) AuNPs at various time points (2 h, 4 h, 6 h).** Results are presented in relative units compared with controls. Data represent the mean ± standard deviation of three individual experiments. p < 0.05.

2’,7’– dichlorofluorescein emission by the macrophage cell lines incubated for various time points demonstrated that both AgNPs and AuNPs were able to generate significant ROS activity in both the cell lines indicating macrophage stimulation. The best results were obtained uptill 4 h of incubation, which declined at 6 h (Figure 6a-d). Here, the comparable ROS generation by both the nanoparticles from non-stimulated cells to mitogen-stimulated cells was observed and indicates towards the immunomodulatory capability of the synthesized metal nanoparticles. The ROS generation by AgNPs was found better when compared to AuNPs which may be due to the small size of AgNPs as these easily penetrate through the cell wall in an appreciable number than AuNPs, which are larger.

The maximum free radical activity was obtained by the *J774* cells (Figure 6a and b) and *THP1 α* cells (Figure 6c and d) uptill 4 h of incubation with NPs.

## Discussion

Phosphorous is an essential mineral nutrient, which plays an important role in plant physiological processes. Plants uses phosphorous in soluble form, thus phosphate solubilizing fungi are the important microbes, which are responsible for conversion from insoluble to soluble state. In the present study, a novel phosphate solubilizing fungal isolate, FUK 29, was isolated and subjected to characterization. The sequence of 18S rRNA of the fungal strain FUK 29 was submitted to GenBank with an accession number of (KF934408). The homology search using BLAST indicated a close genetic relation of the strain FUK 29 with the rRNA sequence of *B. tetramera* (18S: 95% similarity with the reference sequence BankIt1680597 Seq4) in NCBI database. Such a higher identical value confirmed the strain FUK 29 to be *B. tetramera*.

Myconanotechnology, an exotic branch has become a broad field of study involving chemistry, physics, engineering, computing, electronics, energy, agriculture and biomedicine. In the realm of biomedicine, nanotechnology is widely touted as one of the next promising and important approaches to diagnose and treat various ailments [18]. Since chemical and physical methods of metal nanoparticle-synthesis are expensive and involve incorporation of toxic chemicals, the biological synthesis using bacterial, fungal and plant extract sources have been found a preferred option. This owes to their ease of availability, nontoxic nature and quicker synthesis. This prompted us to use this novel fungus *B. tetramera* for the synthesis of AgNPs and AuNPs. The recent reports indicate that the synthesis of AgNPs/AuNPs is based on the surface plasmon resonance involving color alteration [19]. The culture supernatant of selected fungi was used for the synthesis of AgNPs and it is proposed that the reduction of Ag^+^ might be due to the involvement of protein components contributed by the enzyme NADH-reductase. Further, in the UV/VIS absorption, a strong peak located at 380 nm and 570 nm was observed for AgNPs/AuNPs respectively. Such type of UV-visible peak is in accordance with the previous report on assorted metal nanoparticles, which ranged between 6 to 100 nm in size [20].

Average particle size calculated from DLS data was found to be 109.4 nm for AgNP while for AuNP it is 73.82 nm. Furthermore, the SEM image of the synthesized AgNPs/AuNPs validated the formation of spherical, triangular and hexagonal nanoparticles. This indicated the reduction of Ag^+^ to elemental silver (Ag) and Au^+^ to elemental gold (Au). Therefore, the UV/VIS spectra showed for AgNPs/ AuNPs at 380 nm and 570 nm and the spherical images observed in this study for NPs through SEM was in agreement to those reported earlier [21]. Additionally, the synthesized nanoparticles were stable in solution over a time of three months at room temperature. Nano-silver/gold is an effective and a fast-acting microbicide against a broad spectrum of common bacteria and fungi, thus have been utilized in various processes in the medical field [22]. The similar results were obtained in the present study where both the silver and gold nanoparticles exerted high antibacterial activities along with the standard antibiotic (tetracycline) against *Bacillus cereus.* The antibacterial activity of AgNPs/AuNPs is due to the permeability of the cell membrane [23] or formation of free radicals [24] or interaction of AgNPs/AuNPs with the thiol groups of many enzymes thus inactivating them. The efficacy of AgNPs could be attributed to the fact that their larger surface area enabled them to have a better contact with the microorganisms. This is further supported by the revelation that size dependent interaction of silver nanoparticles with bacteria leads to its antibacterial activity [25]. The toxicity of silver ions, though not very clearly understood, could be either due to adhesion to the cell membrane and further penetration inside or by interaction with phosphorus containing compounds like DNA disturbing the replication process or preferably by their attack on the respiratory chain. It has also been suggested, that a strong reaction takes place between the silver ions and thiol groups of vital enzymes thus inactivating them [26]. Some studies also reported that the attachment of the nanoparticles on to the surface of the cell membrane disturbs the permeability and respiration functions of the cell [27]. Earlier experimental evidences have advocated the loss of replication ability by the DNA when treated with silver ions [28]. Beside this, the moderate antifungal activity of AgNPs against *Aspergillus niger* and *Trichoderma* was due to formation of insoluble compounds by inactivation of sulfhydryl groups in the fungal cell wall and disruption of membrane bound enzymes and lipids, which causes cell lysis [29]. The AuNPs did not possess any antifungal activity.

Metal nanoparticles can induce toxicity at various degrees. It is suggested that higher concentrations of silver nanoparticles are toxic and can cause various health problems. AgNPs were found to be significantly more toxic to *THP1 α* cells as compared to *J774* cells*,* which could be attributed to the intrinsic anticancer property of AgNPs. AuNPs, however, possess moderate activity. This might be due to small particle size of AgNPs with enormous specific surface area, which facilitated further expression and dissolution of ions in comparison to AuNPs thus, potentially leading to increased toxicity. AgNPs are highly reactive and exhibit oxidative potential and ability to bind with biomolecules like proteins and DNA, resulting in creating the disturbance in the functioning of biomolecules [30]. Moreover, the maximum free radical activity was attained by the *J774* and *THP1 α* cells uptill 4 h of incubation. Analogous encapsulation efficacy and free radical generation capability of both metal nanoparticles project their use as a potential drug/vaccine delivery vehicle for macrophages as well as also indicate towards their immunomodulatory activity.

The present study also supported the notion as higher doses of silver/gold nanoparticles exhibited the cytotoxicity on both the cell lines used. The results revealed the potential use of biologically synthesized metal nanoparticles as an anti-microbicide [31], occupying its application in agriculture. Moreover, these nanoparticles can also act as immunomodulatory agent alone or in combination with established therapeutic immunomodulatory agents [32]. As these NPs are easily engulfed by the macrophages, they also pose themselves as targeted drug/vaccine delivery vehicle to macrophages thereby a boom for development of a potent chemotherapeutic vehicle for diseases involving macrophages *viz.*, leishmaniasis, tuberculosis etc [33]. Hence, care has to be taken to utilize these nanoparticles in a good, effective and efficient way, understanding its limitations and taking extreme care that it does not cause any harm to an individual or the environment. It can be believed that if utilized properly, silver nanoparticles can be a good friend, but if used haphazardly, they can become a mighty foe.

In future, the bio-conjugated nanomaterials (encapsulation) may lead to enhancement of agricultural productivity for slow release of phosphorus, from soil and fertilizers and its effective uptake by plants. As *B. tetramera* contains acid phosphatase enzyme involved in phosphate solubilization, the bio-nanoparticles prepared from them may display slow release of encapsulated enzyme and hence may enhance phosphate solubilization. The nanoparticles thus prepared may be mixed with fertilizers to enhance the uptake of phosphorus. However, this needs to be validated.

## Conclusion

In the present study among all isolated and well characterized fungal species, *B. tetramera* having efficient phosphate solubilizing ability therefore the culture supernatant FUK 29 was selected for the synthesis of metal AgNPs and AuNPs. It was confirmed that the AgNPs/AuNPs formed were spherical in shape. The AgNPs/AuNPs generated showed the promising antimicrobial agent against both Gram-positive/Gram-negative bacteria and pathogenic fungi also. On the contrary, AuNPs which were found to be nontoxic in cytotoxic assays could be used as a vehicle for drug delivery. However, higher doses of silver/gold nanoparticles exhibited the cytotoxicity on both the cell lines (*J774* and T*HP1α*). The fungal strain FUK 29 used in this study is likely to provide broad-spectrum benefits such as (i) its utility in the solubilization of insoluble phosphate into soluble form, (ii) for used to generation of AgNPs/AuNPs, and (iii) its effectiveness in the implementation of infectious diseases (leishmaniasis, tuberculosis) as drug delivery vehicle as well as in the area of agriculture (antibacterial and antifungal agent).

## Additional file

Additional file 1: Figure S1. Phylogenetic analysis of *Bipolaris tetramera* (KF934408). **Figure S2.** UV-VIS spectrophotometry of nanoparticles. **Figure S3.** DLS spectrum of nanoparticles.
