# Supplementary material for: Antimicrobial and immunomodulatory efficacy of extracellularly synthesized silver and gold nanoparticles by a novel phosphate solubilizing fungus Bipolaris tetramera
Source: BMC Microbiol. 2015 Feb 27;15:52. doi: 10.1186/s12866-015-0391-y (PMC4364495; doi:10.1186/s12866-015-0391-y)
Supplement: Additional file 1: Figure S1. — Phylogenetic analysis of Bipolaris tetramera (KF934408). Figure S2. UV-VIS spectrophotometry of nanoparticles. Figure S3. DLS spectrum of nanoparticles. [file 12866_2015_391_MOESM1_ESM.doc]

**Supplementary Data**


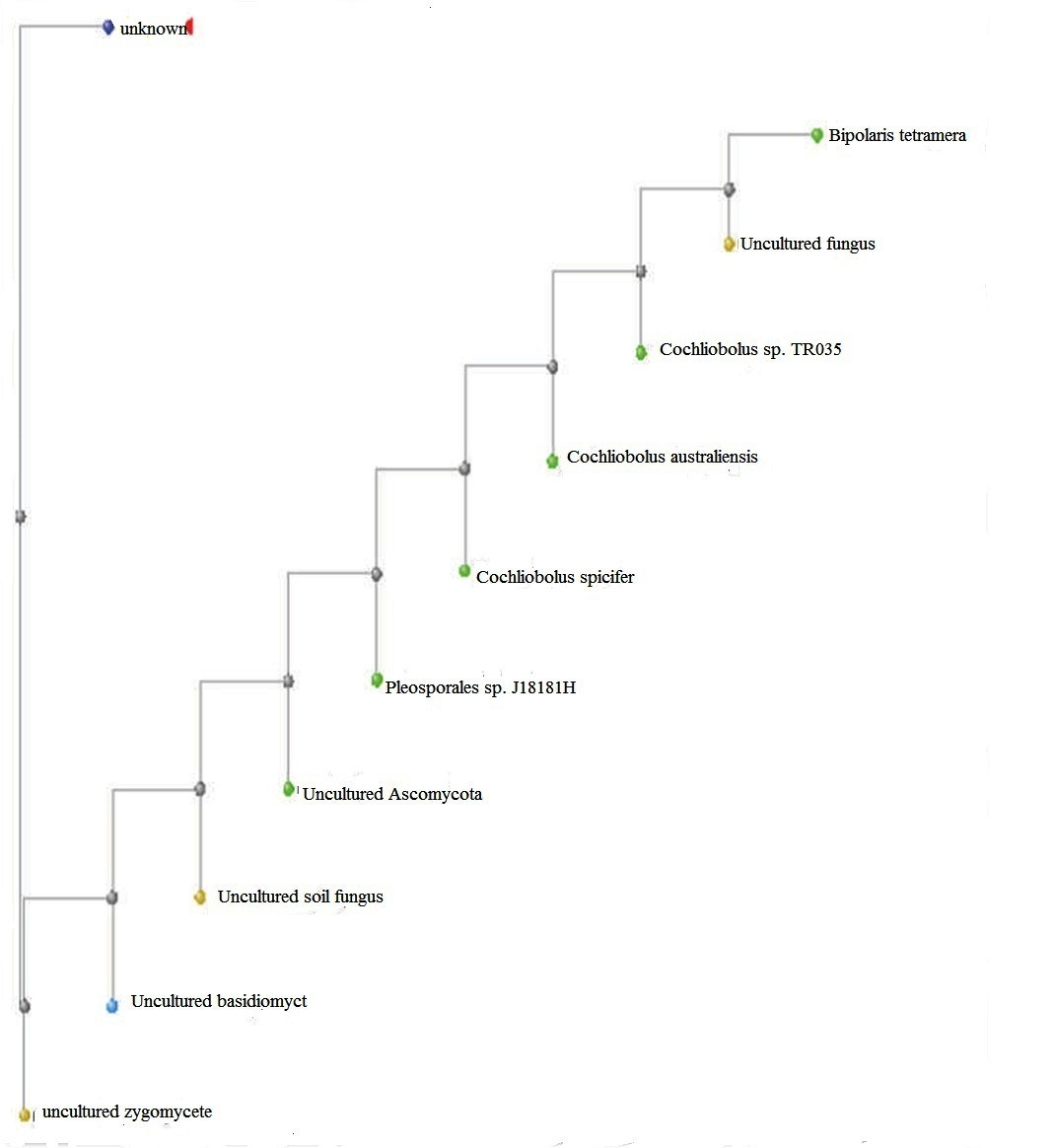


**Figure S1: Phylogenetic analysis of *Bipolaris tetramera* (KF934408)**


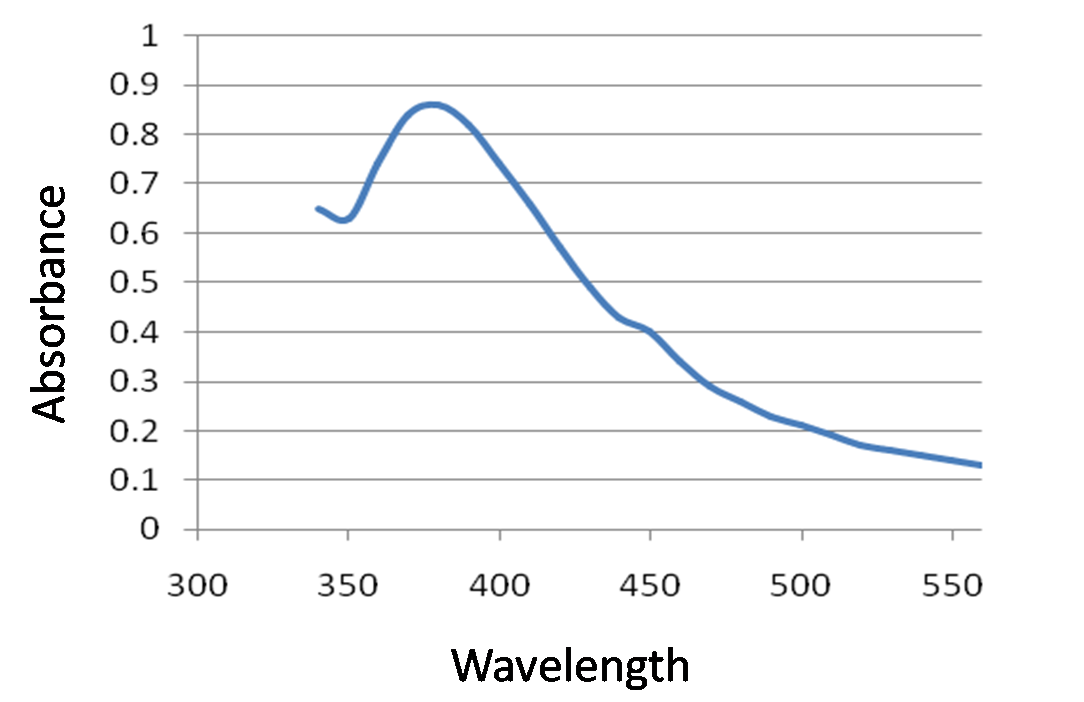
**
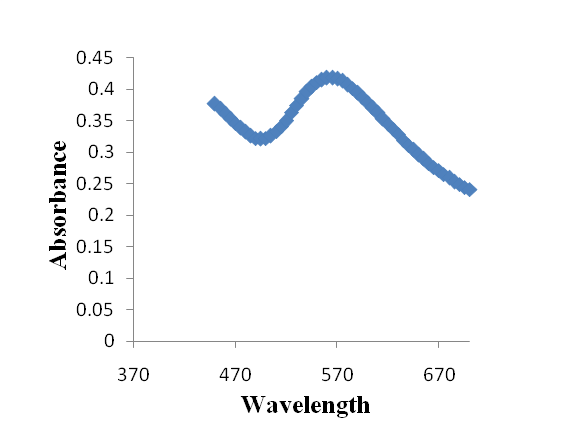
**

1. **(b)**

**Figure S2: UV-VIS spectrophotometry of nanoparticles. (a) AgNP; (b) AuNP**

**
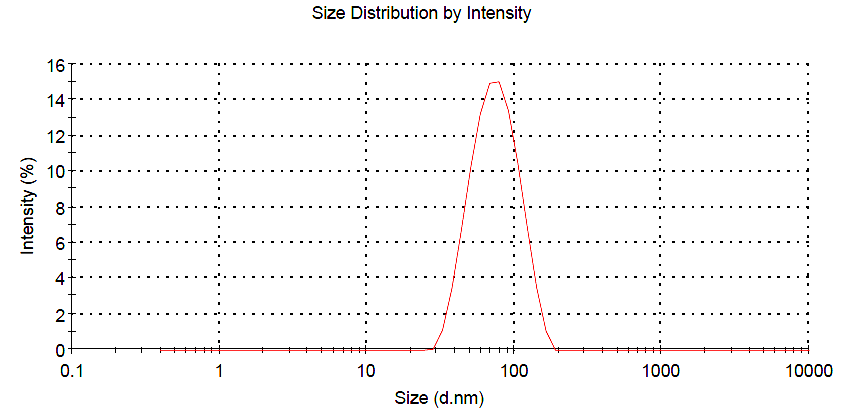

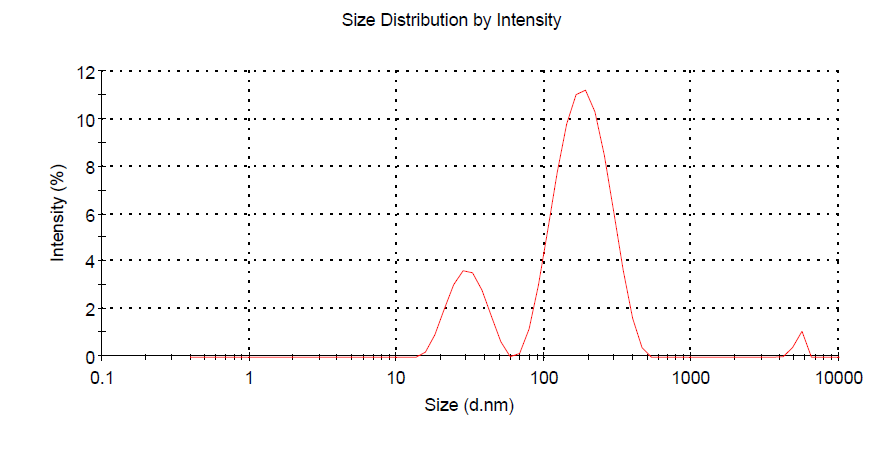
**

**Figure S3: DLS spectrum of nanoparticles. (a) AgNP; (b) AuNP**
